# CD9 in acute myeloid leukemia: Prognostic role and usefulness to target leukemic stem cells

**DOI:** 10.1002/cam4.2007

**Published:** 2019-02-10

**Authors:** Lucas Touzet, Florent Dumezy, Christophe Roumier, Céline Berthon, Claire Bories, Bruno Quesnel, Claude Preudhomme, Thomas Boyer

**Affiliations:** ^1^ Laboratory of Hematology CHU Lille Lille France; ^2^ Department of Hematology CHU Lille Lille France

**Keywords:** acute myeloid leukemia, AML, CD9, leukemia stem cells, LSC, minimal residual disease, multiparametric flow cytometry, prognosis

## Abstract

CD9 is a cell surface protein and belongs to the tetraspanin family. Its role in carcinomagenesis has been widely studied in solid tumors but remains controversial, depending on the cancer type. Although CD9 seems to be associated with unfavorable outcome and disease progression in acute lymphoblastic leukemia (ALL), this marker has not yet been studied in acute myeloid leukemia (AML). First, we explored its prognostic role and its association with biological factors in a cohort of 112 AML patients treated with intensive chemotherapy. CD9 was expressed in 40% of AML and was associated with a favorable outcome (event‐free survival and relapse‐free survival) in univariate (*P* = 0.009 and *P* = 0.048, respectively) and multivariate (*P* = 0.004 and *P* = 0.039, respectively) analyses. Interestingly, CD9 expression was different between the more immature physiologic and AML cells (CD34+CD38−) as it was also expressed in AML on putative leukemic stem cells (LSCs) but not on hematopoietic stem cells (HSCs). Hence, CD9 could be a very relevant marker for minimal residual disease (MRD) monitoring in AML based on LSC targeting.

## 1 INTRODUCTION

Acute myeloid leukemia (AML) is the most common cause of leukemia‐related mortality in adult patients with a 5‐year overall survival (OS) around 40%.^1^ Initially, AML is a clonal disorder of hematopoietic stem cells (HSCs) characterized by an arrest of differentiation with associated proliferation, a subsequent accumulation of blast cells at various stages of incomplete maturation, and a reduced production of healthy hematopoietic elements. Furthermore, AML is a heterogeneous disease at both the phenotypic and molecular levels with an accumulation of successive genetic defects and coexisting clones.^2^ This heterogeneity extends to the leukemic stem cells (LSCs), which are thought to be resistant to intensive chemotherapy based on anthracycline and cytarabine and therefore, mediate disease relapse.^3^ The frequency of these cells at diagnosis is associated with worse outcome in AML.^4^ LSCs are enriched in the CD34+/CD38− cell compartment and these cells are able to reproduce human AML in NOD/SCID mice.^5^ These LSCs are found in this compartment even in *NPM1*‐mutated AML patients which are associated with a CD34‐negative phenotype.^6^

Over the past few years, various markers have been described to better characterize LSC or blast cells from AML with normal cytogenetics (CN‐AML) for minimal residual disease (MRD) monitoring.^7^, ^8^, ^9^, ^10^ Furthermore, targeting MRD by multiparametric flow cytometry (MFC) to monitor treatment efficiency is one of the greatest challenges in the treatment of AML. Recently, we reported a reliable method by MFC to quantify three hematopoietic progenitor populations and the putative LSC compartment by using a combination of antibodies at diagnosis and follow‐up.^11^ However, these markers are not always enough to target LSCs and new strategies are needed, even in conventional MRD by MFC based on leukemia‐associated immunophenotypes (LAIP).

CD9 antigen belongs to the tetraspanin family, which are cell surface proteins clustering in membrane entities called tetraspanin‐enriched microdomains (TEMs).^12^ Tetraspanins contribute to numerous cellular process as cell adhesion, cell activation, or proliferation and have been implicated in several carcinogenous processes such as angiogenesis or metastasis in human cancers.^13^ Among them, CD9 was first known as a tumor suppressor but several studies showed its oncogenic and prometastatic functions,^14^, ^15^, ^16^ suggesting that the different partners of CD9 in TEMs could explain these different roles. In hematological malignancies, CD9 has been mainly studied in acute lymphoblastic leukemia (ALL) where it might promote cancer stem cell‐like properties and dissemination of the disease with CXCR4‐mediated migration.^17^, ^18^ Its role in AML remains controversial:a few studies suggested a pejorative role of CD9 with a decreased OS^19^ and a negative association with t(8;21)(q22;q22)^20^ while another study showed a correlation between CD9 and mutated *NPM1* AML^21^ which are associated with a favorable prognosis. Here, we analyzed the expression of CD9 on AML primary cells and physiologic progenitors, the prognostic role of CD9 on survival in AML patients treated with intensive chemotherapy, its association with classical biological factors and its usefulness to discriminate LSCs from HSCs.

## 2 MATERIALS AND METHODS

### 2.1 Patients

One hundred and twelve patients with AML de novo diagnosed between 2009 and 2016 and treated with intensive chemotherapy were included in this study. Patients with acute promyelocytic leukemia were excluded. Patients were treated with induction chemotherapy (continuous infusion of cytarabine for 7 days with daunorubicin or idarubicine for 3 days) and at least two consolidation courses after complete remission (CR). High‐risk cases (unfavorable cytogenetic or combined genetic risk, patients with early relapse…) underwent hematopoietic stem cell transplantation (HSCT). All patients were treated in the department of Hematology of Lille hospital. Signed informed consent was obtained from each patient in accordance with the declaration of Helsinki. Cytogenetic and molecular risks and CR criteria were determined according to European Leukemia Net recommendations.^22^

### 2.2 Multiparametric flow cytometry

Diagnostic blast cells were obtained from cryopreserved bone marrow (BM) aspirates. MFC results were not different between fresh and frozen cells for three patients (data not shown). Each sample was washed twice in RPMI with 10% fetal bovine serum (FBS) at 37°C then stained for 30 minutes at room temperature with the following antibody panel: anti‐CD9‐PE (clone HI9a, Biolegend), anti‐CD19‐ECD (clone J3‐119, Iotest, Beckman Coulter), anti‐CD33‐PC5.5 (clone D3HL60.251, Iotest, Beckman Coulter), anti‐CD34‐AA700 (clone 581, Iotest, Beckman Coulter), anti‐CD38‐PB (clone LS‐198‐4‐3, Iotest, Beckman Coulter), and anti‐CD45‐KO (clone J.33, Iotest, Beckman Coulter). To study the hematopoietic progenitors and the putative LSC to perform MRD, a second tube with the following panel:anti‐CD36‐FITC (clone FA6‐152, Iotest, Beckman Coulter), anti‐CD9‐PE (clone H19a, Biolegend), anti‐CD19‐ECD (clone J3‐119, Iotest, Beckman Coulter), anti‐CD33‐PC5.5 (clone D3HL60.251, Iotest, Beckman Coulter), anti‐CD90‐APC (clone 5E10, BioLegend), anti‐CD34‐AA700 (clone 581, Iotest, Beckman Coulter), anti‐CD45RA‐APC‐H7 (clone H100, BD Pharmingen), anti‐CD38‐PB (clone LS‐198‐4‐3, Iotest, Beckman Coulter), and anti‐CD45‐KO (clone J.33, Iotest, Beckman Coulter) was used. Data acquisition was performed on a Navios flow cytometer and analyzed with Kaluza software (Beckman Coulter). The sensitivity of the instrument was verified every day for optical alignment, fluidic stability, optical sensitivity using fluorospheres (Flowset targets™, Flowcheck™, Beckman Coulter).

The gating strategy for flow cytometry analysis was then performed as described previously.^11^ Briefly, blast cells were gated as CD45dim/SSClow population and hematogones (CD38++CD19+ phenotype) were excluded from this gate using these two antibodies. CD34 and CD38 positivity were preset on this population and then, P6 (CD34+CD38−), P7 (CD34+CD38dim), and P8 (CD34+CD38+) populations were determined within blast cells. Finally, from the P6 gated cells, the different progenitor populations were determined using CD90 and CD45RA expression.

### 2.3 Statistical analysis

Comparison of CD9 expression and CD9 mean fluorescence intensity (MFI) between AML bone marrows, normal bone marrows, and hematogones was assessed by Kruskal‐Wallis nonparametric test. Differences between the patients negative and positive for CD9 (ie, less or more than 20% of expression on blast cells) on quantitative variables were assessed by Student t test and qualitative variables were compared using chi‐square test.

Quantitative variables associated with either OS, event‐free survival (EFS), and relapse‐free survival (RFS) were tested with the Cox model. OS, EFS, and RFS were then described by the Kaplan‐Meier method. For patients who underwent BM transplantation, survival was censored at the date of transplantation and for patients alive, survival was censored at the date of last known alive. Multivariate analysis was performed with a Cox method:hazard ratios were adjusted on variables with significant pronostic value (*P* < 0.05) for EFS, OS, and RFS.

A *P‐*value < 0.05 (two‐tailed) was considered statistically significant. Statistical analysis was performed using SPSS 22.0 software (IBM, New York, USA).

## 3 RESULTS

### 3.1 CD9 expression on normal and leukemic cells

**Figure 1:**
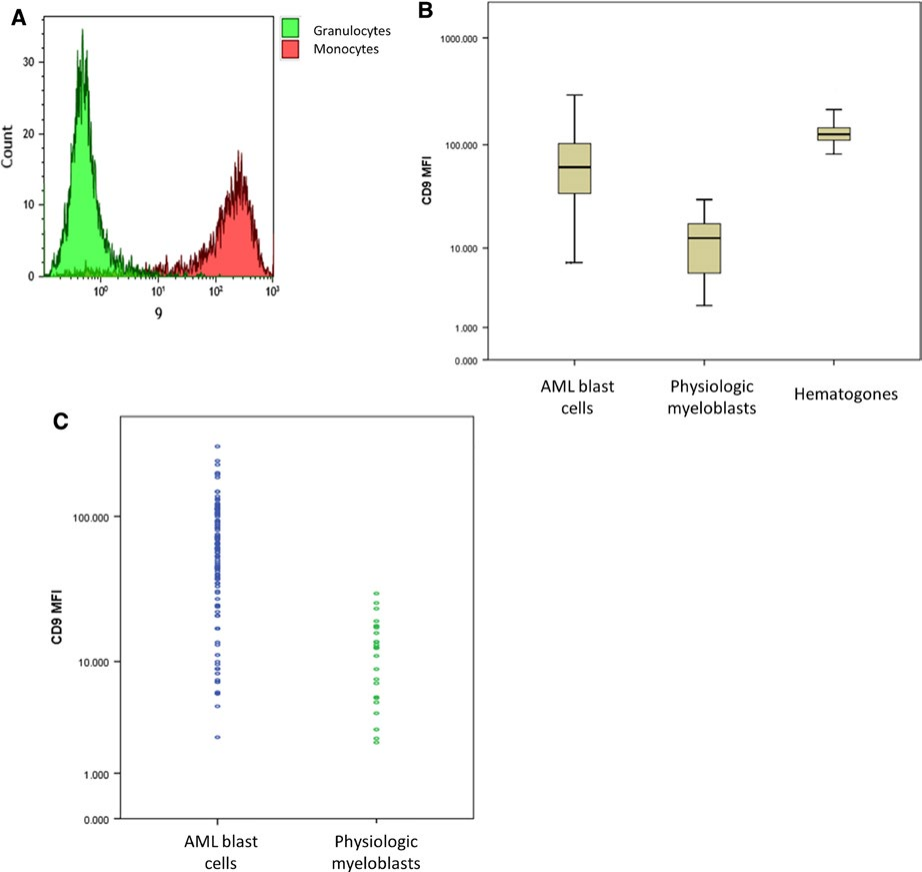
CD9 expression on hematogones, physiologic myeloblasts, and AML blast cells. (A) Expression of CD9 on physiologic cells: granulocytes as a negative control (green) and monocytes (red) as a positive control (B). Comparison of CD9 MFI on blast cells between diagnostic bone marrow from patients with de novo AML (n = 112, normal bone marrow samples (n = 25) and hematogones from normal bone marrow (n = 25) (C). Comparison of CD9 MFI on blast cells between AML and normal bone marrow samples

CD9 expression was observed on monocytes on normal BM and normal peripheral blood, and absence of CD9 expression was observed on granulocytes as previously reported (Figure 1A).^23^ We analyzed CD9 expression on normal hematogones (HTG) (CD38++ CD19+ CD45 dim), on physiologic myeloblasts gated as CD45dim/SSClow cells after exclusion of hematogones from 25 normal BM and on blast cells from 112 AML patients with the same gating. CD9 expression was strong and homogeneous on normal HTG (MFI median = 125, range 81.7 to 256.3), heterogeneous on blast cells (median of expression = 29.8%, range = 0 to 99%, MFI median = 66, ranging from 2.5 to 294), and weak and homogeneous on physiologic myeloblast compartment (median of expression = 6.4%, range = 1.3 to 16.6%, MFI median = 12.6, range = 2.2 to 30.1, Figure 1B). A significant higher expression of CD9 was found on malignant blast cells compared to physiologic myeloblasts considering percentages of expression and MFI levels (Figure 1C, Kruskal‐Wallis nonparametric test *P* < 0.001). Thus, we could consider expression of CD9 as a frequent leukemia‐associated immunophenotype (LAIP) in our study.

### 3.2 CD9 and prognostic factors in AML

**Figure 2:**
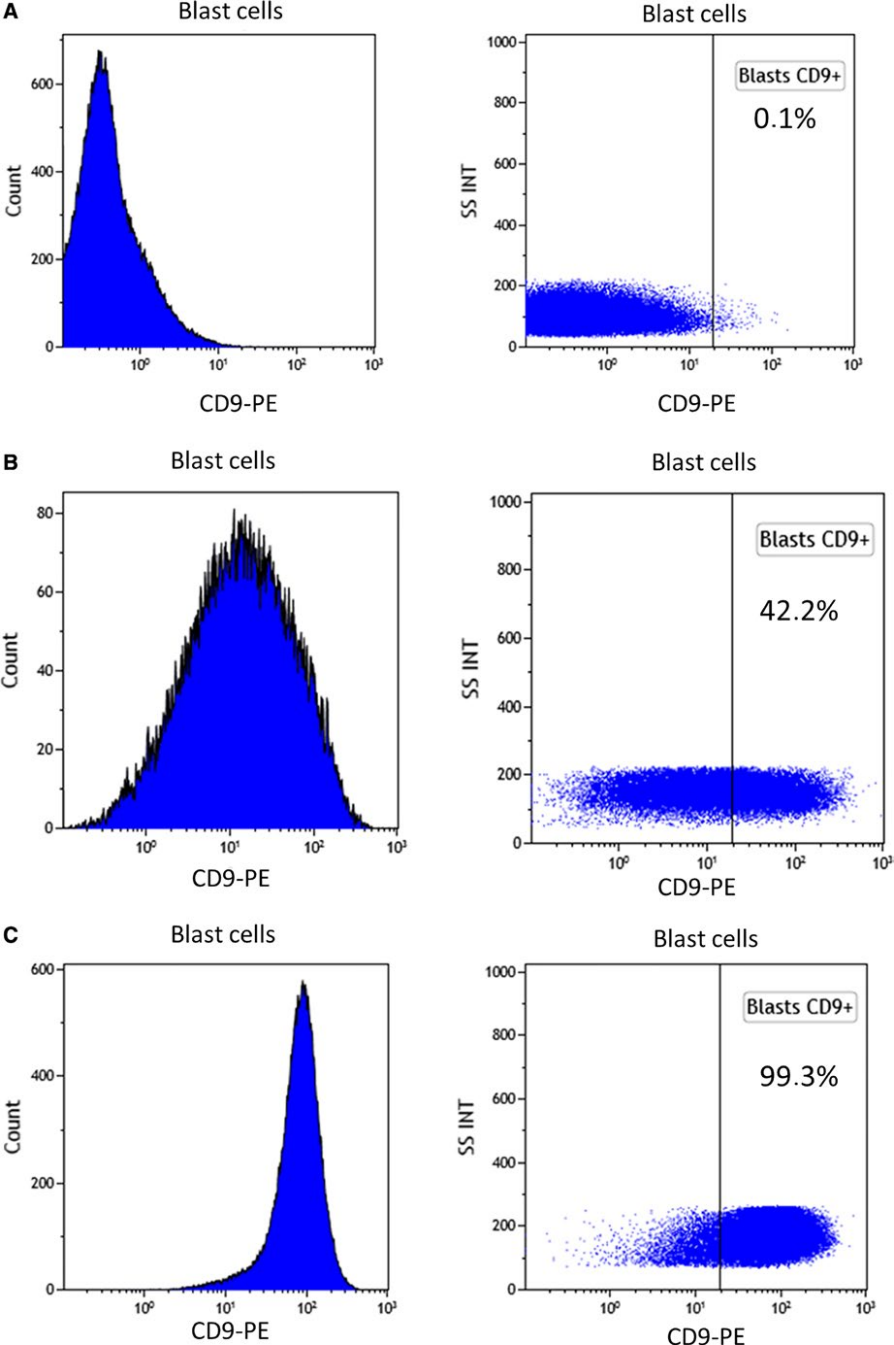
AML samples have varying CD9 expression on blast cells. Representative examples of MFI histograms of different types of AML according to CD9 expression: (A). Example of negative CD9 blast expression (0.1%) (B). Example of intermediate CD9 blast expression (42.2%) (C). Example of high CD9 blast expression (99.3%)

One hundred and twelve patients were included in our study with ages ranging from 21 to 78 years. First, we observed three types of CD9 expression profile:weak or negative (Figure 2A), intermediate with heterogeneous positive expression (Figure 2B) and strong and homogenous expression (Figure 2C). Then, we compared patient characteristics according to CD9 expression (Table 1). Expression of CD9 was found in 45 of 112 patients (40%) and there was no association with sex, age, white blood count (WBC), FAB type, hemoglobin level, platelet count, peripheral blood (PB) blast cells percentage, bone marrow (BM) blast cells percentage, cytogenetic risk, *FLT3‐ITD* and *NPM1* mutations. CD9‐positive AML tended to include more AML with *NPM1* mutation (*P* = 0.09). There was no association of CD9 expression with classical LAIP (CD7 expression, lack of CD13/CD33 expression, CD33 overexpression…).

**Table 1:** Patient characteristics

	All patients (n = 112)	<20% CD9− blasts (n = 67)	>20% CD9+ blasts (n = 45)	*P*‐values
Gender (M/F)	61/51	36/31	25/20	0.45
Age (y)^a^	51.9 (20.9‐77.6)	51.3 (20.9‐74.1)	52.4 (20.5‐77.6)	0.45
WBC (10^9^/L)	68 (1‐405)	67 (1‐405)	72 (3‐300)	0.67
PB blast cells (%)	44 (0‐99)	45 (0‐99)	43 (2‐97)	0.92
BM blast cells (%)	65 (5‐98)	66 (5‐98)	64 (20‐97)	0.58
FAB type n (%)				0.24
M0	4 (4)	2 (3)	2 (3)	
M1	18 (16)	11 (16)	7 (16)	
M2	29 (26)	23 (35)	6 (13)	
M4	31 (28)	14 (21)	17 (38)	
M5	16 (14)	8 (12)	8 (18)	
M6	2 (2)	2 (3)	0 (0)	
ND	11 (10)	7 (10)	4 (9)	
Hemoglobin level (g/dL)^a^	9.3 (3‐15)	9 (4‐14)	10 (3‐15)	0.45
Platelet count (10^9^/L)^a^	82 (7‐803)	80 (12‐803)	82 (7‐341)	0.98
Cytogenetic risk n (%)				<0.001
Favorable	14 (13)	6 (9)	8 (18)	
Intermediate	78 (77)	47 (70)	31 (70)	
Adverse	16 (17)	12 (18)	4 (9)	
ND	4 (4)	2 (3)	2 (3)	
*FLT3‐ITD* n (%)	31 (28)	17 (25)	14 (31)	0.42
*NPM1* n (%)	37 (33)	19 (28)	18 (40)	0.09
*CEBPa *dm n (%)	4 (4)	4 (4)	0 (0)	0.22

WBC, white blood cell count; M, male; F, female; PB, peripheral blood; BM, bone marrow; ND, not done.

a Median with range in parenthesis.

### 3.3 Prognostic role of CD9 in AML

We used univariate analysis to evaluate the following parameters for EFS, OS, and RFS: age, WBC count, hemoglobin level, platelet count, cytogenetic risk group, *FLT3‐ITD *status, *NPM1* mutational status, and CD9 expression. A multivariate model for EFS, OS, and RFS was used for all variables with a *P*‐value < 0.05. All the results are summarized in Table 2. As expected, cytogenetics was the most powerful variable (*P* < 0.001) for EFS, OS, and RFS.

**Table 2:** Univariate and multivariate analyses for EFS, OS, and RFS according to CD9 expression and other biological parameters

EFS	Univariate HR (range) *P*‐value	Multivariate HR (range) *P*‐value
Age	1.024 (1.006‐1.043) *P* = 0.01	1.012 (0.99‐1.03) *P* = 0.27
FAB type	1.002 (0.822‐1.222) *P* = 0.98	NI
Cytogenetic risk	4.28 (2.46‐7.45) *P* < 0.001	*P* = 4.9 (2.65‐9.06) ***P* < 0.001**
*FLT3‐ITD*	2.4 (1.31‐4.32) *P* = 0.004	3.32 (1.68‐6.59) ***P* = 0.001**
*NPM1* mutation	0.82 (0.41‐1.62) *P* = 0.19	NI
CD9 + blasts	0.46 (0.26‐0.82) *P* = 0.009	0.35 (0.17‐0.72) ***P* = 0.004**

OS	Univariate HR (range) *P*‐value	Multivariate HR (range) *P*‐value
Age	1.044 (1.017‐1.071) *P* = 0.001	1.03 (0.998‐1.064) *P* = 0.07
WBC count (10^9^/L)	1.004 (1‐1.007) *P* = 0.035	0.999 (0.994‐1.004) *P* = 0.62
FAB type	0.964 (0.773‐1.278) *P* = 0.96	NI
Cytogenetic risk	4.57 (2.37‐8.8) *P* < 0.001	4.53 (2.16‐9.45) ***P* < 0.001**
*FLT3‐ITD*	2.9 (1.39‐6.04) *P* = 0.004	1.538 (1.208‐2.39) *P* = 0.23
*NPM1* mutation	0.74 (0.33‐1.69) *P* = 0.38	NI
CD9 + blasts	0.613 (0.309‐1.217) *P* = 0.16	NI

RFS	Univariate HR (range) *P*‐value	Multivariate HR (range) *P*‐value
Age	1.035 (1.011‐1.061) *P* = 0.004	1.044 (1.012‐1.077) ***P* = 0.007**
WBC count (10^9^/L)	1.004 (1.001‐1.008) *P* = 0.02	0.998 (0.992‐1.004) *P* = 0.47
FAB type	1.084 (0.85‐1.38) *P* = 0.53	NI
Cytogenetic risk	3.001 (1.54‐5.89) *P* = 0.001	3.29 (1.52‐7.13) ***P* = 0.003**
*FLT3‐ITD*	3.2 (1.5‐6.85) *P* = 0.003	2.55 (1.07‐6.05) ***P* = 0.034**
*NPM1* mutation	0.96 (0.42‐2.21) *P* = 0.57	NI
CD9 + blasts	0.496 (0.244‐1.008) *P* = 0.048	0.405 (0.172‐0.955) ***P* = 0.039**

*P*‐values number marked in bold are significant in multivariate analysis

**Figure 3:**
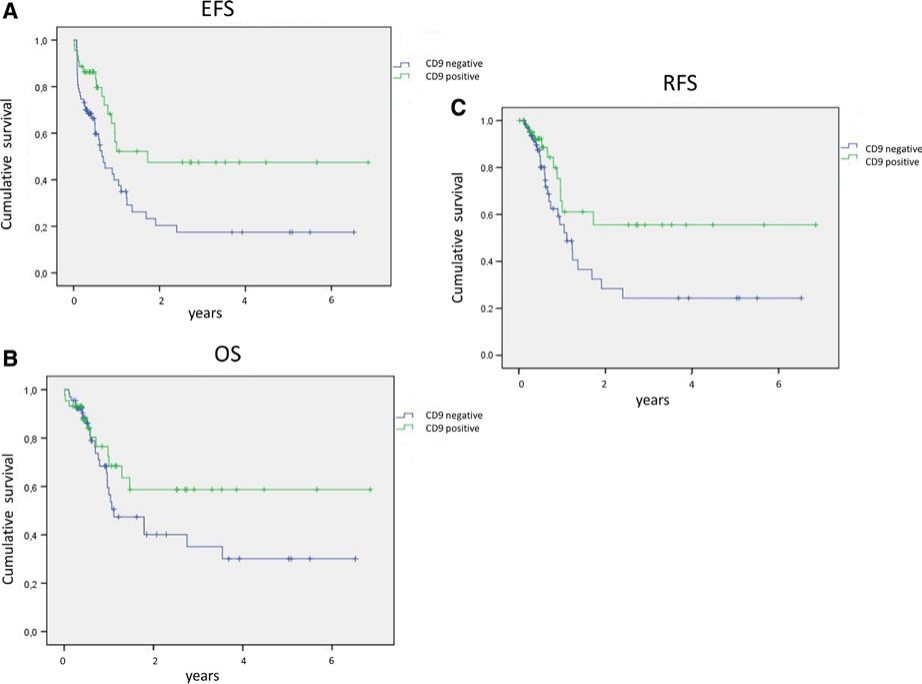
CD9 expression on blast cells is associated with favorable outcome in AML survival curves of (A). EFS (B). OS, and (C). RFS stratified by CD9 expression (negative: blue, positive: green) on blast cells at diagnosis of AML

For EFS, in univariate analysis, age (*P* = 0.01), cytogenetic risk (*P* < 0.001), *FLT3‐ITD* (*P* = 0.004), and CD9 expression (*P* = 0.009) (Figure 3A) had a significant prognostic role. In our multivariate model, only *FLT3‐ITD* (*P* = 0.001), cytogenetic risk (*P* < 0.001), and CD9 expression (*P* = 0.004) were independent prognostic factors: CD9 expression on blast cells positively affected EFS, especially for CN‐AML patients (data not shown).

For OS, in univariate analysis, CD9 expression on blast cells had no significant impact (*P* = 0.16) (Figure 3B).

For RFS, in univariate analysis, age (*P* = 0.004), WBC count (*P* = 0.02), cytogenetic risk (*P* = 0.001), *FLT3‐ITD* (*P* = 0.003), and CD9 expression (*P* = 0.048) (Figure 3C) had significant prognostic value. In our multivariate model, only WBC count had no significant value but CD9 positivity did (*P* = 0.039).

On the contrary, CD9 MFI had no impact on survival (EFS: *P* = 0.92; OS: *P* = 0.98, RFS; *P* = 0.16).

Then, percentage of CD9‐positive cells was associated in this study with significant better EFS and RFS in univariate and multivariate analyses.

### 3.4 CD9 is expressed on LSC and not on the more immature progenitors

We then investigated CD9 expression on normal hematopoietic progenitors (HSCs) and on their leukemic counterparts (LSCs) to use CD9 as a marker of MRD in the most immature compartment. We studied progenitor cell populations based on the expression of the following antigens: HSC (CD34+CD38−CD45RA−CD90+), Multipotent Progenitors (MPP: CD34+CD38−CD45RA−CD90dim), Lymphoid‐Primed Multipotent Progenitors (LMPP: CD34+CD38−CD45RA+CD90−), and the putative LSCs (CD34+CD38−) which harbored various patterns of expression of CD90 and CD45RA.^10^ These phenotypic definitions of progenitor cells have been previously verified in functional assays.^24^

**Figure 4:**
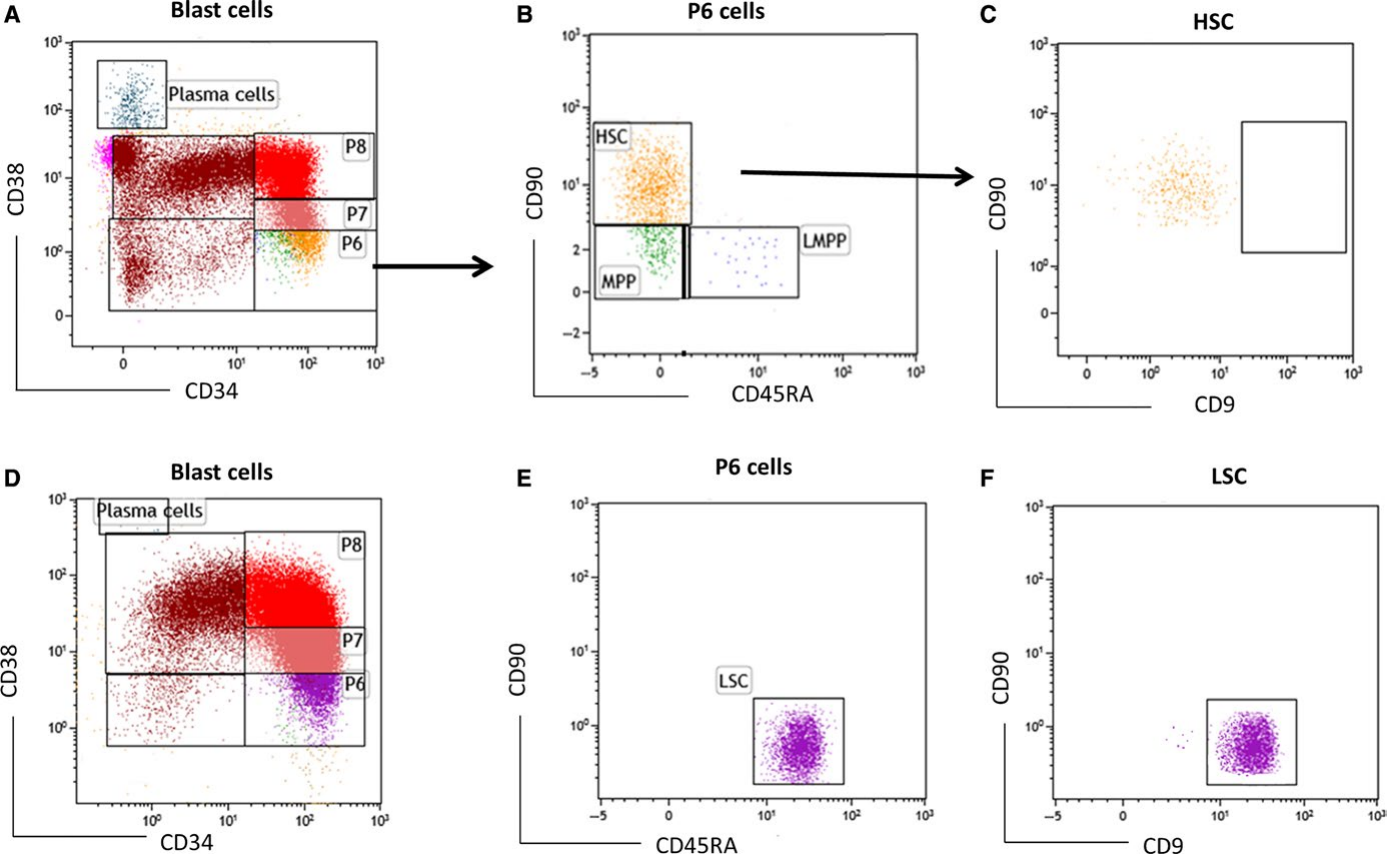
CD9 is not expressed on normal CD34+CD38− physiologic cells but is expressed on phenotypically most immature AML cells A. Blast cells are separated using CD34 and CD38 expression into several progenitors:P6 (CD34+CD38−), P7 (CD34+CD38dim) and P8 (CD34+CD38+) cell compartments B. Determination of HSC, MPP, LMPP in a normal bone marrow sample C. CD9 is not expressed on HSC D. Blast cells are separated using CD34 and CD38 expression into P6, P7, and P8 cell compartments E. Determination of HSC, MPP, and putative LSC (CD34+CD38−CD90dimCD45RA+) in a bone marrow sample from a patient at AML diagnosis F. CD9 is expressed on putative LSCs at similar MFI level compared to AML blast cells

**Figure 5:**
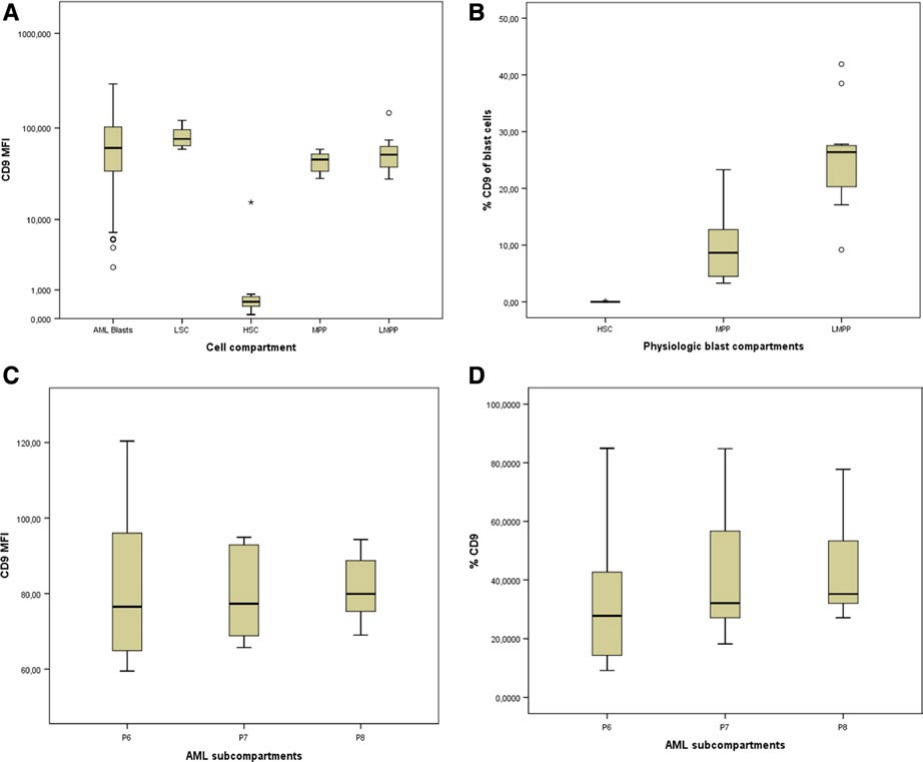
Expression of CD9 in physiologic blast compartments and AML subcompartments. (A) MFI values on AML blast cells, LSC, HSC, MPP, and LMPP (B). Percentage of CD9+ cells on HSC, MPP, and LMPP (C). MFI values and (D). Percentage of CD9+ cells in CD34+38+ (P6), CD34+CD38dim (P7), and CD34+CD38+ (P8) AML cells

CD9 expression on these populations was studied in 17 normal BM and 17 BM from CD9+ AML patients. Interestingly, the expression of CD9 was always positive on LSCs and always negative on HSCs from normal BM (Figures 4 and 5A). We observed a similar CD9 MFI level on LSC compared to AML blast cells (MFI on LSCs, median = 76 and MFI on blast cells, median = 66, *P* = 0.11, Figure 5A). On normal bone marrow progenitors, CD9 appeared at the MPP stage and was observed on LMPP with similar MFI values (Figure 5A). Nevertheless, the percentage of CD9‐positive cells was higher at the LMPP stage (Figure 5B). On the contrary, CD9 expression and CD9 MFI were steady in all AML subcompartments such as CD34+CD38− (P6), CD34+CD38dim (P7), and CD34+CD38+ (P8) AML cells (Figure 5C,D). Hence, CD9 could help to easily discriminate HSCs from LSCs and then, could be a very interesting marker to monitor MRD by MFC on progenitors cell compartment in AML.

## 4 DISCUSSION

Acute myeloid leukemia is an aggressive condition and chemotherapy regimens need to be adapted to the patient's disease. New prognostic tools are then important, especially for CN‐AML patients, to refine the prognosis. One of the most interesting measurement of treatment response is MRD monitoring. Detection by MFC of markers expressed in blast cells is one of the most studied field over the past few years but identification of a cell antigen only expressed in AML (and not in physiologic myeloblasts) has not been successful so far.

In our study, CD9 is expressed in 40% of AML and, considering its expression at a significant level on LSCs, it should be investigated when these cells are studied by the MRD strategy that we described previously.^11^ In ALL, CD9 seems to be associated with cancer stem cell properties too and is involved in leukemic progression.^25^ Interestingly, CD9 antigen level of expression is identical between LSCs and AML blast cells and is not expressed on normal HSCs. These findings are particularly relevant as the majority of antigens associated with LSCs (ie, TIM3, CLL1, and CD244) are less expressed on these cells compared to bulk cells and are frequently coexpressed on normal HSCs and progenitors.^26^ Recently, Coustan‐Smith et al published a study on MRD monitoring in AML by MFC and, interestingly, they found an overexpression of CD9 on blast cells (in 30% of AML cases) compared to physiologic myeloblasts.^27^ Furthermore, they showed that CD9 was abnormally overexpressed on CD34+CD38− AML cells at diagnosis and relapse of 10 paired samples, thus corroborating the MFC strategy on LSCs that we used in this study. We investigated CD9 expression on progenitors from 17 normal BM and 17 AML BM so to strengthen the conclusions on LSCs, more AML, and normal samples, need to be studied.

With these first results, targeting CD9 in AML seems to be an interesting approach to eliminate LSCs and prevent relapses with monoclonal antibodies or even chimeric antigen receptor (CAR) T‐cell therapy. Anti‐CD9 has already been used in vitro and could induce apoptosis of Jurkat cells and inhibition of proliferation of B‐ALL cells.^25^, ^28^ CD9 is also widely expressed on monocytes and depletion of these cells could be interesting in AML as they are implicated in disease progression by producing interleukin‐1.^29^

In our study, we demonstrated a favorable role of CD9 on AML prognosis, especially on EFS and RFS in univariate and multivariate analyses. This favorable role was related to the percentage of CD9+ blast cells but was not highlighted according to the MFI values. Some of the patients in this cohort had a weak (higher than granulocytes) but uniform expression of CD9 and then, were considered CD9 positive. Conversely, in other patients, a few blast cells had a bright CD9 MFI but were considered CD9 negative because of the small number of those cells. Therefore, the density of CD9 antigen at cell surface is not directly linked to RFS or EFS. However, the expression of CD9 on blast membranes reflects probably changes in the cellular properties of these cells. How this expression could participate to a better chemosensitivity should be investigated (tetraspanin molecules have been largely reported to participate to various cellular processes like cell domiciliation or quiescence which could be related to this chemosensitivity).

Furthermore, this is a retrospective study and the prognostic value of CD9 needs to be determined on larger cohorts in prospective studies.

Liu et al showed a significant expression of CD9 in AML with *NPM1* mutation and, therefore, a favorable prognosis, which is consistent with our findings. In contrast, CD9 seems to be associated with a worse prognosis in ALL treated with intensive chemotherapy, as it was demonstrated in a cohort of 87 patients.^30^ These different results are probably explained by the association of CD9 in TEMs with different partners in ALL and AML. It could be interesting to characterize these partners and then, study the functional role of CD9 in AML.

In conclusion, CD9 is abberantly expressed of 40% of AML in our study and is associated with favorable outcome. Furthermore, this marker is expressed on LSCs and not on HSCs when the blast cells are CD9 positive. It would be of interest to perform MRD with the LSC targeting that we used here in prospective studies to better assess treatment response and then, to predict relapse.
